# Navigation of Dual Identities Among Health Care Professionals Transitioning Between Clinical Practice and Research Roles: Scoping Review Protocol

**DOI:** 10.2196/76490

**Published:** 2026-05-15

**Authors:** Shahad Al-Tikriti, Clare Kennedy, Karima Abubakr, Mary Higgins

**Affiliations:** 1 Department of Obstetrics and Gynaecology, Rotunda Hospital, Parnell Street, Dublin, D01 W5P9, Ireland, +353 1 817 1700; 2 Department of Obstetrics and Gynaecology, National Maternity Hospital, Dublin, Ireland

**Keywords:** professional transition, clinician-researcher, medical education, career development, professional identity, scoping review

## Abstract

**Background:**

The transition from clinical practice to research represents a significant yet under-studied career pivot for health care professionals. Despite the growing emphasis on research integration in health care careers and increasing numbers of clinicians pursuing advanced research degrees, the transition experience remains poorly understood. This protocol outlines a scoping review aimed at examining the nuanced experiences of clinicians moving between full-time clinical practice and research roles, exploring the challenges, adjustments, and impacts on professional identity, decision-making, and career trajectories.

**Objective:**

This scoping review aims to systematically map and synthesize existing literature on health care professionals’ experiences in transitioning between clinical practice and research roles.

**Methods:**

This scoping review will follow the JBI methodology for scoping reviews and validated frameworks. Comprehensive searches will be conducted across 8 databases: PubMed, Ovid, Scopus, Embase, CINAHL, PsycInfo, Sage Journals, and Web of Science. Studies will be eligible for inclusion if they (1) focus on health care professionals providing direct patient care (physicians, nurses, midwives, pharmacists, dieticians, and other allied health professionals), and medical students, (2) examine transitions between clinical practice and research roles, (3) report empirical data, and (4) are published in English from 1980 onward. Two independent reviewers (SA-T and CK) will screen titles, abstracts, and full texts. A rigorous data extraction tool will be developed and piloted. Qualitative thematic analysis will be used to synthesize findings.

**Results:**

This protocol was registered with the Open Science Framework in January 2024. This scoping review will be conducted as part of SAT’s MD (doctor of medicine, University College Dublin, commenced July 2023) conducted at the National Maternity Hospital. As of January 2026, pilot database searches identified approximately 3000 potentially relevant studies. Preliminary analysis of a subset of randomly chosen studies was performed as a pilot test to refine the search strategy and inclusion or exclusion criteria. Full data collection will commence following protocol acceptance, with complete results expected to be submitted for publication in 2026.

**Conclusions:**

By synthesizing existing knowledge on clinical-research transitions, this review will aim to inform evidence-based support mechanisms, guide institutional policies, and enhance the training experience for the growing number of health care professionals navigating this career pathway. The findings will provide a foundation for a larger mixed methods study addressing identified knowledge gaps.

## Introduction

### Background and Significance

Professional transitions represent critical junctures where individuals experience discontinuity in their established routines and must develop new behaviors to adjust to changed circumstances [1,2]. In health care contexts, transitions are increasingly recognized as periods of vulnerability yet also opportunities for transformative learning and professional growth [3,4]. While medical education literature has extensively explored 3 traditional transition periods—preclinical to clinical training, undergraduate to postgraduate training, and trainee to independent practitioner [5]—a fourth significant transition between clinical practice and research roles remains comparatively under-studied.

Throughout this manuscript, we use the term “clinician-researchers” to refer to all health care professionals who engage in both clinical practice and research activities, encompassing physicians, nurses, midwives, allied health care professionals, and medical students in intercalated programs such as MD-PhD programs. When citing specific studies, we retain authors’ original terminology (eg, clinical academics, physician-scientists, and nurse-scientists) to maintain fidelity to the source literature.

Health care systems worldwide increasingly emphasize the integration of research with clinical practice, reflected in the substantial rise in clinicians pursuing higher research degrees [6]. In the United Kingdom alone, the number of clinical academics has grown by 43% over the past decade, with similar trends observed across Europe, North America, and Australia [7]. The MD-PhD and clinician-scientist pathways have become formalized career tracks, with dedicated funding streams and institutional support mechanisms [8,9]. Despite this growth, limited attention has been paid to the unique challenges faced by professionals navigating between these distinct domains.

The transition between clinical practice and research represents more than a simple change in workplace or responsibilities; it involves shifting between fundamentally different epistemological frameworks, accountability structures, and measures of success [10]. Preliminary evidence suggests this transition can profoundly impact professional identity, creating tensions between clinician and researcher roles [11,12]. Understanding these experiences is crucial not only for the well-being of the individuals involved but also for optimizing the development of clinician-researchers who can effectively bridge the implementation gap between research and practice [13].

### Theoretical Framework

This study will be informed by two complementary theoretical frameworks: the transition theory by Bridges [14], which conceptualizes transitions as 3-phase processes involving endings, neutral zones, and new beginnings; and the theory of professional identity transitions by Ibarra [15], which emphasizes the provisional selves’ individuals construct when moving between professional domains. Together, these frameworks provide conceptual tools for understanding how health care professionals navigate the emotional, cognitive, and practical aspects of moving between clinical and research roles.

To clarify our conceptual framework, we explicitly distinguish between two related but distinct concepts: “Transition” refers to the temporal process of moving between clinical and research roles (informed by the transition theory by Bridges [14]), characterized by phases of ending (leaving established clinical practice), neutral zone (the liminal period of adjustment), and new beginning (establishing oneself in the research domain of reentering clinical practice). “Dual identities” describes the identity state that may result from these transitions, specifically the simultaneous holding of both clinician and researcher professional identities. This state may involve integration of both identities and ongoing tension between them.

The transition to research often requires individuals to relinquish established clinical identities temporarily or permanently, navigate an uncertain middle period where competence may be questioned, and gradually construct new professional identities integrating elements of both domains [16]. Some individuals successfully develop integrated dual identities, while others may experience persistent role conflict or ultimately abandon one role in favor of the other. Recent empirical work has demonstrated the practical application of this conceptual distinction in examining full-time clinical-research transitions [17].

### Rationale for a Scoping Review

A preliminary search of PROSPERO, MEDLINE, the Cochrane Database of Systematic Reviews, and JBI Evidence Synthesis was conducted in September 2023, revealing no existing or in-progress reviews on this specific topic. While literature on clinician-researcher career pathways exists, it is fragmented across medical education, health care management, professional development, and academic medicine domains. A scoping review methodology is therefore appropriate to map this diverse literature, identify key concepts and gaps, and provide the foundation for more focused inquiry [18,19].

The findings from this review will inform the development of evidence-based support mechanisms for health care professionals undergoing this transition and guide institutional policies aimed at facilitating successful integration of clinical and research career paths. Additionally, it will establish the groundwork for a larger mixed methods study examining the lived experience of clinician-researcher transitions.

### Review Questions and Objectives

This scoping review aims to answer the following questions:

- What is the experience of health care professionals transitioning from full-time clinical practice to full-time research roles?
- What is the experience of health care professionals returning to clinical practice after a period of full-time research?
- What is the experience of medical students undertaking intercalated research programs in comparison with that of health care professionals?

Specific objectives include the following: identifying common challenges and facilitating factors in clinician-researcher transitions; exploring impacts on professional identity, well-being, and career trajectories; mapping existing support mechanisms and their reported effectiveness; identifying gaps in the current literature to inform future research priorities; and comparing transition experiences across career stages by examining differences between medical students undertaking intercalated research programs and established health care professionals transitioning to research roles.

## Methods

This scoping review will adhere to the JBI methodology for scoping reviews [20,21], which builds upon the framework developed by Arksey and O’Malley [21] and enhanced by Levac et al [22]. The protocol has been developed in accordance with the PRISMA-ScR (Preferred Reporting Items for Systematic Reviews and Meta-Analyses Extension for Scoping Reviews) [23] and registered with the Open Science Framework [24].

### Eligibility Criteria

The proposed eligibility criteria are defined using the population, concept, and context framework. This review will include empirical studies focusing on the transition of health care professionals between full-time clinical practice and full-time research roles. Detailed criteria and eligible source types are summarized in Table 1.

**Table 1.** Population, concept, and context framework defining inclusion and exclusion criteria for the scoping review.

	Inclusion criteria	Exclusion criteria
Population	Health care professionals providing direct patient care; physicians, nurses, and nurse practitioners, midwives, pharmacists, dieticians or nutritionists, allied health care professionals, and medical students	Veterinarians (different professional context from human health care) Dentists (dental clinical academic training pathways and research degree frameworks differ substantially from medical pathways) Studies where professional backgrounds cannot be clearly identified
Concept	Transitions from full-time clinical practice to full-time research roles Transitions from full-time research to full-time clinical practice Dual clinical-research identities Impact on professional identity, well-being, and career trajectories Transition challenges and facilitating factors Support mechanisms	Studies focusing solely on research productivity without examining transition experiences Studies examining only clinical training transitions without research component Studies focused exclusively on research methodology without addressing transition experience
Context	Transitions for higher degrees (eg, MD, PhD, and MSc) Academic medical centers and university teaching hospitals Graduate education and research environments Any country or health care system	Studies published before 1980 Studies not published in English
Sources	Primary empirical research (qualitative, quantitative, and mixed methods) Systematic reviews with primary synthesis of transition experiences	Opinion pieces without empirical data Theoretical papers without empirical data Conference abstracts without full text Letters to the editor without data Book reviews News articles Narrative reviews without systematic methodology

### Search Strategy

A proposed comprehensive search strategy has been developed in consultation with a health sciences librarian. The strategy will be tailored to each database while maintaining conceptual consistency.

### Information Sources

A comprehensive search will be conducted across 8 electronic databases, as detailed in Textbox 1.

Textbox 1. **Electronic databases to be searched.**

- PubMed
- Ovid
- Scopus
- Embase
- CINAHL
- PsycInfo
- Sage Journals
- Web of Science

Gray literature sources will be searched through ProQuest Dissertations and Theses Global for relevant unpublished theses and dissertations. We will also search for policy documents and guidance from organizations supporting clinician-researchers training (eg, Health Service Executive and Royal College of Physicians of Ireland training policies).

Additionally, citation chasing will be used in both backward citation chasing (hand-searching reference lists of all included studies and relevant systematic reviews) and forward citation chasing (using Google Scholar and Web of Science to identify studies that have cited our included studies. This bidirectional approach will maximize identification of relevant literature beyond database searches.

### Search Terms and Strategy

An initial limited search of MEDLINE was conducted to identify relevant keywords and index terms. The search strategy combines 3 conceptual blocks covering population, transition, and research or education terms. The detailed search strategy for PubMed (MEDLINE) is presented in Table 2. This strategy will be adapted for each included database.

**Table 2.** Proposed search strategy for PubMed (MEDLINE).

Search no.	Query	Filters	Results, n
1	“Students, Medical”[Mesh] OR “Nurses”[Mesh] OR “Physicians”[Mesh] OR “Pharmacists”[Mesh] OR “Nutritionists”[Mesh] OR “Nurse Midwives”[Mesh]	—^a^	347,627
2	Transition* OR “role transition” OR “role change” OR “dual role” OR “intercalated program*”	—	612,971
3	“Education, Graduate”[Mesh] OR “md-phd program*” OR “foundation year” OR “out of program*” OR “return to practice”	—	104,529
4	#1 AND #2 AND #3	—	616
5	(“veterinary” [Subheading]) OR “Dentists”[Mesh] OR “Dentistry”[Mesh]	—	869,145
6	#4 NOT #5	From 1980 to 2023	609

a Not applicable; date filters were applied to the final combined search only (search 6) to avoid redundancy.

### Study Selection

Following the search, all identified citations will be collated and uploaded into Rayyan screening software (Qatar Computing Research Institute), and duplicates will be removed. Titles and abstracts will then be screened by 2 independent reviewers against the inclusion criteria. Potentially relevant sources will be retrieved in full, and their citation details will be imported into Rayyan.

The full text of selected citations will be assessed in detail against the inclusion criteria by 2 independent reviewers. Reasons for exclusion of full-text studies that do not meet the inclusion criteria will be recorded and reported. Any disagreements between reviewers at each stage of the selection process will be resolved through discussion or by involving a third reviewer. The results of the search and selection process will be reported in full using a PRISMA-ScR flow diagram.

### Data Extraction

Data will be extracted from included papers by 2 independent reviewers using a standardized data extraction tool developed for this review. The tool will be designed to extract data relevant to the review questions and will capture the following:

- Study characteristics (author, year, country, design, and methodology)
- Participant characteristics (profession, career stage, and sample size)
- Transition context (type of transition, degree pursued, and duration)
- Key findings related to the following:
  - Transition challenges
  - Facilitating factors
  - Impact on professional identity
  - Support mechanisms
  - Recommendations for practice

The proposed data extraction tool is outlined in Table 3.

The data extraction tool will be tested on 5 studies by both reviewers to ensure consistency and comprehensiveness before proceeding with full extraction. Modifications to the extraction tool will be detailed in the final review. Any disagreements between reviewers will be resolved through discussion or with a third reviewer. Authors of papers will be contacted to request missing or additional data where required.

**Table 3.** Proposed data extraction tool for included studies.

Item	Details
Author	Primary author identification
Publication year	Year of study publication
Country of origin	Geographic location of study
Study design	Methodological approach used
Aim of study	Primary research objective
Themes	Key thematic findings
Journal	Publication venue
Participants (type)	Health care professional categories
Sample size	Number of participants
Concept (stage of transition)	Specific transition phase examined
Context (eg, MD, MSc, and PhD)	Type of graduate program or degree
Data collection method	Research methodology used
Summary of key findings	Primary results and conclusions
Relevant references	Supporting literature cited

### Data Analysis and Presentation

In accordance with established scoping review methodology (JBI Manual for Evidence Synthesis [20] and Arksey and O’Malley framework [21]), formal quality appraisal of included studies is not being conducted, as the purpose of this review will be to map the breadth of existing literature rather than to synthesize evidence for clinical recommendations. However, key methodological characteristics of included studies (study design, sample size, data collection methods, and analytical approach) will be reported descriptively in the results to provide transparency about the evidence base.

### Descriptive Analysis

Descriptive analysis will be applied to characterize the included studies. Results will first be presented in tabular form, including the following: distribution of studies by year, country, and methodology; participant characteristics (profession and career stage); types of transitions studied; and methodological characteristics of included studies.

Visual representations (charts and maps) will be used to illustrate the following: geographical distribution of research, professional backgrounds of participants, methodological approaches used, and chronological trends in publication.

### Thematic Analysis

A thematic analysis approach will be used to synthesize findings across studies [25]. This will involve the following: familiarization with the data, generating initial codes (distinguishing between “transition process” codes and “dual identity” codes as per our conceptual framework), searching for themes, reviewing themes, defining and naming themes, and producing the analysis.

Themes will be organized according to the review questions and may include the following: emotional and psychological aspects of transition, professional identity challenges and adaptations (including formation of dual identities), structural and institutional factors, temporal aspects (pretransition, during transition, and posttransition), differences across professional groups, and effective support mechanisms.

The analysis will specifically identify both barriers and facilitators to successful transitions, with attention to how these may vary by profession, career stage, and institutional context.

### Managing Heterogeneity Across Professions and Settings

To address the diversity of professions, career stages, and settings represented in the included studies, our synthesis will use three complementary approaches.

First, matrix mapping—we will create visual matrices categorizing studies by professional background (physicians or medical students, nurses, and allied health professionals) and transition type (clinical-to-research, during education programs, research-to-clinical, and multiple transitions). This will enable identification of patterns and gaps across professions and career stages.

Second, thematic synthesis across diversity—while we will note profession-specific and career stage–specific nuances where they emerge, our primary analysis will identify overarching themes that transcend professional boundaries. We hypothesize that transition experiences share common elements (eg, identity challenges, time management, membership needs, and psychological impacts) regardless of specific profession, while also acknowledging unique contextual factors that may be profession- or setting-specific.

Third, comparative subanalysis—where sufficient data exist, we will conduct comparative analysis between professional groups and transition types to explicitly identify similarities and differences in transition experience. This will help determine whether certain challenges or facilitators are universal across all clinicians-researchers or specific to professional contexts.

This multilayered approach will balance the need to capture diverse experiences while ensuring meaningful synthesis of findings.

### Evidence Gap Mapping

Areas where evidence is limited or lacking will be explicitly identified using an evidence gap map. This will highlight the following: professional groups underrepresented in the literature, aspects of the transition experience requiring further research, methodological limitations of existing studies, and geographical regions with limited research.

### Stakeholder Consultation

To enhance the relevance and applicability of findings, a stakeholder consultation phase will be incorporated following preliminary analysis. This will involve the following: identification of key stakeholder groups (current clinical academics, program directors, and students), virtual consultation workshops to present preliminary findings, structured feedback on interpretation and implications, and integration of stakeholder perspectives into final analysis.

This approach aligns with best practices in scoping review methodology and ensures findings resonate with the experiences of those navigating these transitions.

## Results

This protocol was registered with the Open Science Framework [24].

A comprehensive search will be conducted across 8 electronic databases (PubMed, Ovid, Scopus, Embase, CINAHL, PsycInfo, Sage Journals, and Web of Science). Gray literature sources will be searched through ProQuest Dissertations and Theses Global and relevant organizational policy documents from organizations supporting clinician-research training.

Pilot database searches were conducted in 2024 to test and refine the search strategy and inclusion or exclusion criteria. These pilot searches identified approximately 3000 potentially relevant studies across all databases. Preliminary analysis of a sample of these studies was performed as a pilot test to refine the search strategy and inclusion or exclusion criteria and to refine the data extraction tool.

The data extraction tool (Table 3) has been developed and piloted on 5 randomly selected studies to ensure consistency between reviewers. Minor refinements were made to enhance clarity and completeness. The scoping review is currently in the protocol development phase.

Full data collection will commence following acceptance of this protocol. The complete scoping review process, including systematic searching, screening, data extraction, analysis, and stakeholder consultation, is expected to be completed within 12 months of protocol acceptance. Complete results are expected to be submitted for publication in 2026.

**Figure 1.**
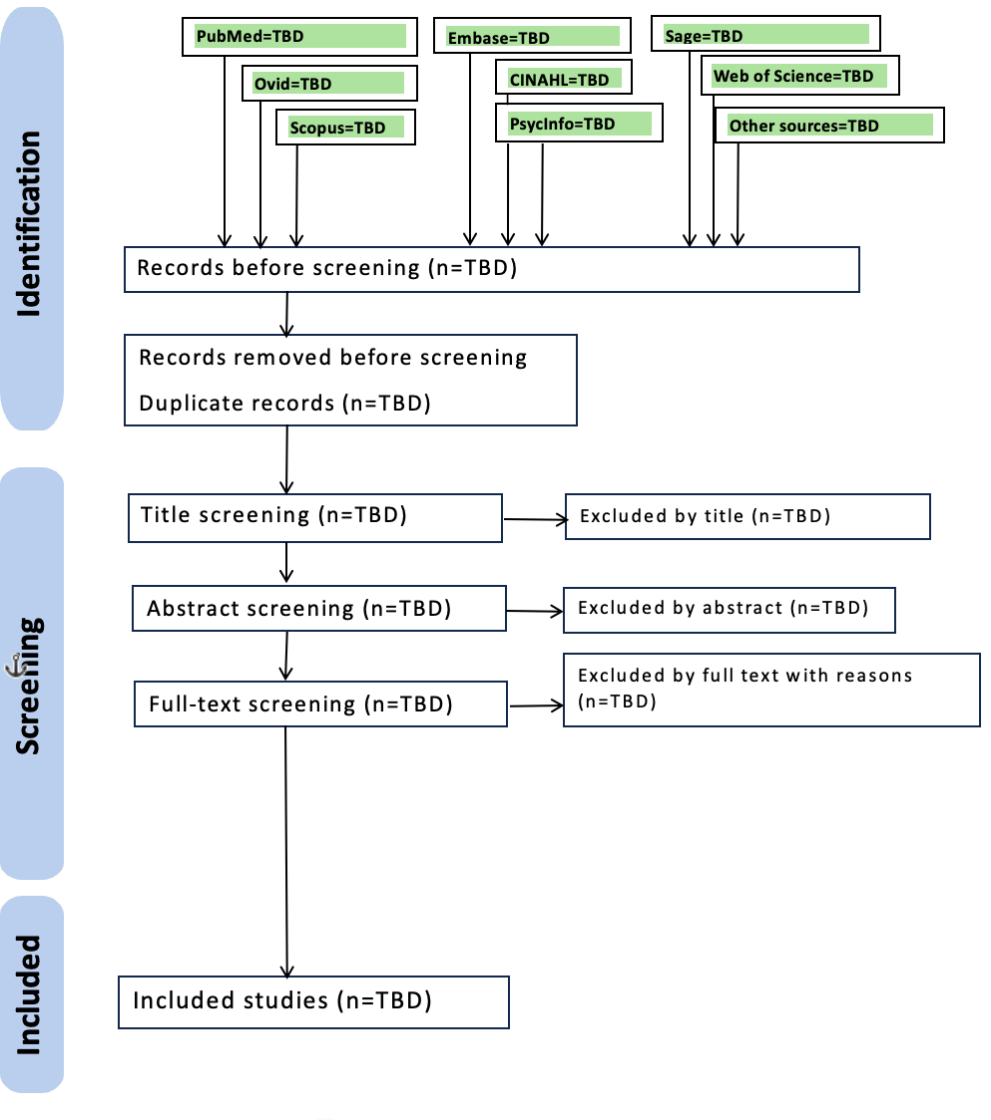
PRISMA-ScR (Preferred Reporting Items for Systematic Reviews and Meta-Analyses Extension for Scoping Reviews) flow diagram showing anticipated study selection process for the scoping review protocol. Note: pilot database searches conducted in 2024 identified approximately 3000 potentially relevant records. This diagram represents the anticipated study selection process that will be implemented following protocol acceptance. TBD: to be determined upon completion of the full review.

Figure 1 presents the anticipated PRISMA-ScR flow diagram structure that will be used to report the final study selection process.

## Discussion

### Anticipated Findings

Pilot database searches conducted in 2024 identified approximately 3000 potentially relevant records across 8 databases. To refine the search strategy and test inclusion criteria, a subset of these records was screened and reviewed. This pilot work revealed several preliminary patterns that inform our expectations for the full scoping review. The sampled studies appeared to focus predominantly on the structure and outcomes of clinician-researcher programs, examining program design, research productivity, and career outcomes rather than the subjective experiences of individuals undergoing these transitions. Furthermore, the pilot screening suggested a relative scarcity of research examining the transition from established full-time clinical practice to full-time research roles and back again. Very few sampled studies appeared to explore the lived experience of the transition itself: the emotional, psychological, and identity-related aspects of moving between these fundamentally different professional domains. This pilot analysis suggests that while programmatic and structural aspects of clinician-research pathways may be well documented, the personal experience of navigating these transitions may remain under-studied—a hypothesis that the full scoping review will test rigorously.

### Comparison to Prior Work

This scoping review will differ from existing literature in several important ways. While sampled studies appear to focus on clinician-researcher career pathways, research productivity, and program structures, this review will explicitly focus on the transition experience from the perspective of the individual. Additionally, the review will address a gap in current knowledge by prioritizing studies that report on the challenges, facilitators, psychological impacts, and identity adjustments experienced during clinician-researcher transitions. The inclusion of diverse health care professionals beyond physicians—including nursing, midwives, allied health professionals, and medical students in intercalated programs—will provide a broader perspective compared to previous reviews that focused predominantly on a single professional group. The explicit conceptual distinction between “transition” as a temporal process and “dual identities” as an identity state provides a theoretical framework that will enable clear categorization and synthesis of transition-related findings. This approach will enable more nuanced synthesis of findings related to both the process of transitioning and the outcome of holding multiple professional identities.

### Strengths and Limitations

This review uses several methodological strengths that enhance its rigor and applicability. The comprehensive search strategy spans 8 electronic databases plus gray literature sources, maximizing identification of relevant studies while minimizing publication bias. The inclusion of diverse health care professions will enable comparative insights and enhance generalizability beyond any single professional group. Adherence to established scoping review guidelines from the JBI and PRISMA-ScR ensures methodological rigor and transparency. The incorporation of stakeholder consultation following preliminary analysis will enhance the relevance and practical applicability of findings for those directly affected by these transitions. The clear conceptual framework distinguishing transition processes from identity states provides theoretical grounding for the synthesis. Finally, the multilayered analytical approach, including matrix mapping and comparative subanalysis, is designed to manage the anticipated heterogeneity across professions, career stages, and settings while still enabling meaningful synthesis of findings.

Several limitations should be acknowledged. The exclusion of non-English language publications may limit geographical representation and potentially miss important cultural perspectives on clinician-researcher transitions. While gray literature searching will partially mitigate this concern, the focus on published literature may still miss valuable insights from unpublished sources or ongoing initiatives. The anticipated heterogeneity in how transitions are conceptualized and studied across different professions and contexts may complicate synthesis, though our analytical framework is designed to address this challenge. Finally, the qualitative nature of many anticipated studies may limit statistical generalizability, though this approach will provide the depth of understanding necessary to capture the nuanced experiences that are the focus of this review.

### Future Directions and Dissemination

This scoping review constitutes the first phase of a larger mixed methods study exploring clinician-researcher transitions. Findings will inform the development of subsequent empirical studies designed to address identified knowledge gaps. Specifically, we will conduct a qualitative study using semistructured interviews to explore lived experiences of clinician-researchers, informed by themes identified in this review. Subsequently, we will develop a survey instrument to quantify challenges and support needs across a larger and more diverse population, building on the qualitative findings to ensure the instrument captures relevant constructs. Ultimately, these foundational studies will inform the design and testing of targeted support mechanisms through an intervention study, creating an evidence-based approach to supporting health care professionals through these critical transitions.

Results from this scoping review will be disseminated through multiple channels to maximize impact across different stakeholder groups. Findings will be published in a peer-reviewed open access journal to ensure accessibility to researchers and clinicians. Presentations at relevant national and international conferences will facilitate dialogue with the medical education and health care workforce communities. Policy briefings will be developed and shared with relevant institutions and professional organizations to inform institutional support structures. Finally, a practical guidance document will be created for health care professionals considering or currently navigating this transition.

### Conclusions

This scoping review protocol establishes a rigorous methodological approach to systematically mapping an under-studied but increasingly important career transition in health care. Upon completion, this review will comprehensively synthesize existing knowledge on how health care professionals experience the shift between clinical practice and research roles, providing the foundational evidence base necessary to develop targeted support mechanisms. The review will illuminate both the challenges that make these transitions difficult and the factors that facilitate successful navigation of this career pathway. This protocol represents the first phase of a larger programmatic research trajectory examining clinician-researcher transitions. Following completion of the scoping review, findings will inform the design of a qualitative study using semistructured interviews to explore lived experiences of clinician-researchers in depth. Subsequently, a survey instrument will be developed to quantify challenges and support needs across a larger population, building on both the scoping review findings and qualitative insights. This progressive expansion, from mapping existing literature to in-depth qualitative exploration to large-scale quantitative measurement, will create a comprehensive evidence base about clinician-researcher transitions across different health care professions and settings. The significance of this work extends beyond individual career trajectories, with broader implications for health care systems and research capacity. By progressively building understanding through complementary methodological approaches, this programmatic research aims to guide policymakers, program directors, and clinical supervisors in developing effective support mechanisms for health care professionals navigating this critical career juncture. Understanding and supporting successful clinician-researcher transitions strengthens the vital bridge between clinical practice and research, ultimately enhancing capacity for translational research and evidence-based innovation in health care.

## Abbreviations

PRISMA-ScR: Preferred Reporting Items for Systematic Reviews and Meta-Analyses Extension for Scoping Reviews
